# Cultural Factors of Suicidality

**DOI:** 10.1192/j.eurpsy.2022.109

**Published:** 2022-09-01

**Authors:** L. Küey

**Affiliations:** Istanbul Bilgi University, Department For Psychology, Istanbul, Turkey

**Keywords:** a personal act, a socio-cultural phenomenon, suicidal behavior

## Abstract

Suicidal behavior is a complex human behavior expressed in a spectrum of various acts. From a suicidal gesture to a completed suicide, all reflect a cry for help and need clinical and scientific attention. The process ending up with suicidal act is shaped by multi-factors, including the socio-cultural ones. Suicide is indeed related to a deep feeling of hopelessness; not to have any control over their lives and circumstances except than deciding to stay alive or dead, and so related to serious psychopathologies, as depressive and substance use disorders. Hence, it is frequently seen as a personal act or as a question of individual decision. But since Durkheim‘s ground breaking work, which still inspires suicide researchers, the cultural factors behind this socially determined phenomenon have been widely discussed. Suicide is totally a personal act and a fully socio-cultural phenomenon. The cultural factors of suicidality are among the social determinants of health/ill health. Epidemiological evidence and cross-cultural comparisons show huge differences in suicide rates across countries and even between regions of same countries, and these are constant differences. Furthermore, even the definition of suicide is effected by the social circumstances. Certain socio-cultural patterns shape how and when people commit suicide; i.e., these patterns have decreasing or increasing effect on suicide rates, which provides basis for suicide prevention. Likewise, social solidarity, high group integration and collective sensitivity may have preventive effects. This brings us to the discussion of the effects of big social turmoil or wars or pandemics on suicidal behavior.

**Disclosure:**

No significant relationships.

